# Mechanical and optical properties of a borosilicate glass used to improve the finishing of 3Y-TZP restorations

**DOI:** 10.1590/1807-3107bor-2024.vol38.0077

**Published:** 2024-09-02

**Authors:** Ana Carolina da SILVA, Camila da Silva RODRIGUES, Juliana de Freitas Gouveia SILVA, Clarice Ferreira SABINO, Gilmar Patrocínio THIM, Renata Marques de Melo MARINHO, Tiago Moreira Bastos CAMPOS

**Affiliations:** (a) Universidade Estadual Paulista– Unesp, Institute of Science and Technology, Department of Dental Materials and Prosthodontics, São José dos Campos, SP, Brazil.; (b) Instituto Tecnológico de Aeronáutica – ITA, Physics Department, São José dos Campos, SP,Brazil.

**Keywords:** Boron, Glass, Mechanical Test, Ceramics

## Abstract

Borosilicate glass was developed to enhance the mechanical behavior and smoothness of dental zirconia as an alternative to conventional glaze. This study assessed the mechanical and optical properties of 3 mol% yttria-stabilized tetragonal zirconia polycrystal (3Y-TZP) coated with borosilicate glass or a commercial glaze fired for an extended period of time. Disc-shaped 3Y-TZP zirconia specimens (Zpex, Tosoh) were sintered at 1550°C for 2 hours. The specimens were divided into three groups: as-sintered (control, C); commercial glaze (G); and borosilicate glass (SL). The glaze and borosilicate glass were applied over the zirconia and fired for 20 minutes at 950°C and 1200°C, respectively. Biaxial flexural strength, fractography, X-ray diffraction (XRD), roughness (Ra and Rz), fracture toughness (Vickers indentation method), color difference (∆E_00_), and translucency (TP_00_) analyses were conducted. The t-test or the one-way ANOVA and Tukey’s tests were used to analyze the data (α = 0.05). Flexural strength data were subjected to the Weibull analysis. The SL group exhibited the highest flexural strength (1025.8 MPa), whereas the C (859.41 MPa) and G (816.0 MPa) groups exhibited similar values. The SL group also had the highest characteristic strength. The fracture origin in all groups was on the zirconia surface. XRD analysis revealed that the specimens from the SL group contained tetragonal, cubic, and monoclinic phases. The SL group presented the lowest surface roughness. Fracture toughness in the SL group was lower than in the C group, but similar to that observed in the G group. The translucency and color differences observed in the G and SL groups were similar. Borosilicate glass enhanced the flexural strength of 3Y-TZP, promoted the smoothest surface, and exhibited optical properties similar to those of the glaze.

## Introduction

The biomimetic and mechanical properties of dental materials significantly impact the success of oral rehabilitations. Dental zirconia, commonly employed as a restorative material, particularly in monolithic restorations, often necessitates additional laboratory finishing procedures such as stains, glaze application, or polishing with rubber points to achieve a natural appearance. Beyond their aesthetic significance, these techniques may also influence the clinical and mechanical behavior of dental ceramics.^1 - 3^

Glazing enhances the clinical outcome of dental ceramic restorations by smoothing the surface, resulting in a glossy and natural final appearance.^4^ Previous studies have indicated that glazing does not impact the fatigue behavior of 3% mol yttrium-stabilized tetragonal zirconia polycrystals (3Y-TZP).^2 , 5^ However, concerns regarding opposing tooth wear and internal flaws have been raised in relation to the glaze layer.^6 - 8^ The weak interaction between zirconia and glaze creates a region of fragility, potentially leading to delamination or the formation of critical defects.^8^

Extended glaze firings have been proposed as an alternative method for finishing ceramic restorations.^9 - 11^ Studies have shown that extended glaze firing can enhance flexural strength and reduce tensile residual stresses at the surface of porcelain-veneered zirconia.^11^ This technique involves adjusting the glaze firing process by prolonging the holding time at the maximum temperature, typically from 1 to approximately 15 minutes.^9 - 11^ According to Callister and Rethwisch,^12^ when glasses are heated to a temperature close to that of their softening point, stresses are alleviated within approximately 15 minutes, known as the “annealing point”.

In an effort to enhance the mechanical properties of dental zirconia without compromising its optical characteristics, novel compositions of glass materials for finishing have been developed.^8 , 13^ During the development of these glasses, various ion concentrations can be incorporated into their compositions to achieve specific final characteristics. This is possible because the glass structure does not depend on a particular stoichiometry.^14^ Glasses that are boron-doped are commonly referred to as Pyrex. ^15^ When utilized as a dopant in a glass matrix, boron (B) has the ability to alter the intrinsic properties of the material. Consequently, borosilicate glass forms a stable material at elevated temperatures with a low coefficient of thermal expansion (CTE; 3.25 × 10^-6^ K^-1^).^15 , 16^

The application of a low-CTE glass, such as borosilicate glass, onto zirconia (CTE: 10.8× 10^6^ K^-1^) tends to enhance the mechanical performance of the set.^17^ Borosilicate glass application on ceramics is a well-established method for inducing compression stress at the surface, thereby favoring the mechanical properties of the material.^18^ The borosilicate glass used in this study represents an advancement over the experimental glaze developed by Campos et al.^8^ In previous studies, the thermally compatible glaze was calcinated at 1530°C, whereas the borosilicate glass was fired at 1,200°C, a temperature close to the glaze and stain characterization range (850–950°C).^8 , 13^ The biaxial flexural strength provided by the borosilicate layer exhibited values 14% higher than that of the experimental glaze calcinated at 1530°C. In contrast, conventional glazing of zirconia does not contribute to an increase in the mechanical strength of the materials.^2 , 5^

Nevertheless, the application method for the developed borosilicate glass described herein is similar to the glaze slurry method, yet it has the capability to generate an even smoother surface and enhance the mechanical strength of zirconia. This innovation represents a more user-friendly application technique, making it easier for dental technicians.

Hence, a borosilicate glass was developed for application as an alternative finishing material for 3Y-TZP zirconia, aiming to enhance mechanical properties in comparison to both glaze and no coating. Accordingly, the aim of this study was to assess the mechanical and optical properties of 3Y-TZP coated with an experimental borosilicate glass and a commercial glaze subjected to an extended firing time. As-sintered specimens served as controls. Complementary analyses, encompassing optical properties, roughness, crystalline content, and fracture toughness, were conducted. The tested hypothesis posited that the zirconia specimens coated with borosilicate glass would have better mechanical properties and reduced roughness compared with those from the other study groups, without altering their optical properties.

## Methodology

This in vitro study assessed the impact of surface treatment factors (experimental borosilicate glass, commercial glaze, or as-sintered) on various outcomes, including flexural strength, roughness, translucency, color change, and toughness. Table 1 provides details on the materials utilized in this study, including their composition, manufacturers, and batch numbers.

**Table 1:** . Description of the materials used in the study.

Materials	Composition	Manufacturer	Batch number
Zirconia 3Y-TZP	90.4–94.5 wt% ZrO_2_; 4–6 wt% Y_2_O_3_; 1.5–2.5 wt% HfO_2_; 0–0.3 wt%Al_2_O_3_; 0 wt% Er_2_O_3_; 0–0.3 wt% Fe_2_O_3_	Zpex (ZPex, Tosoh Corporation, Tokyo, Japan)	ZY308567B
Glassy-based material applied by brush	Special low fusing glaze material to create a silky matte and sealed surface	Vita Akzent (Akz 25), VITA Zahnfabrik, Bad Sackingen, Germany	38030 powder 22601 building liquid
Borosilicate glass	SiO_2_ -68%; Al_2_O_3_ -1.1%; CaO 8.93%; Na_2_O -14.54%, MgO – 3.41%, K_2_O -0.59% and B_2_O_3_ – 4%.	Developed by authors	-
Propylene glycol solution	C_3_H_8_O_2_	Labsynth, Diadema, São Paulo, Brazil	178730

### Preparing the borosilicate glass powder

The borosilicate glass was obtained using a silicic acid source, following the methodology developed by Campos et al.^19^ The corresponding salts were incorporated, and the final composition of the borosilicate glass comprised SiO_2_ (68%), Al_2_O_3_ (1.1%), K_2_O (0.59%), Na_2_O (14.54%), CaO (8.93%), MgO (3.41%), and B_2_O_3_ (4%). The glass underwent a heat treatment in an oven at 100°C for 24 hours, followed by calcination at 600°C for 5 hours. Subsequent to this process, the material was ground in an alumina mortar and sieved through a 200-mesh screen.

### Zirconia disc preparation

A total of 0.85 g of 3Y-TZP zirconia powder (ZPex, Tosoh Corporation, Tokyo, Japan) underwent uniaxial pressing for 30 s at 1148 kgf. A tungsten carbide cylindrical matrix measuring 9 cm in height and 5 cm in diameter and having a retractable base, an internal piston, and an internal hole for disc pressing was used to produce discs measuring 15 mm in diameter by 1.5 mm in thickness (n = 90). The discs were subsequently sintered at 1550°C for 2 hours (Sirona inFire HTC speed), and were then randomly distributed into three groups (n = 30) based on the subsequent surface treatment applied to them: commercial glaze with extended firing (G group), experimental borosilicate glass (SL group), or as-sintered, representing the control specimens (C group). The final dimensions of the specimens were 12 mm in diameter by 1.2 mm in thickness (ISO 6872).

### Glaze and borosilicate glass application

For the G group, 0.18 g of glaze powder (Vita Akzent GLAZE, VITA Zahnfabrik, Bad Säckingen, Germany) was mixed with 5 drops of building liquid (Vita Akzent FLUID, VITA Zahnfabrik, Bad Säckingen, Germany) until achieving a homogeneous consistency. Subsequently, a thin layer of the mixture was applied to one surface of the zirconia discs using a fine-tipped brush. The discs underwent firing in a vacuum furnace using the following settings: 400°C initial temperature, 5-minute heating time, 80°C/min temperature elevation rate, 950°C final temperature, and a 20-minute dwell time at the final temperature.

For the SL group, 0.20 g of borosilicate glass powder was mixed with 0.20 g of propylene glycol (P.A.-A.C.-S, Labsynth, Diadema, Brazil). This mixture was then applied with a brush following the same procedure as that described for the glaze groups. The discs underwent firing with the following settings: 400°C initial temperature, 5-minute heating time, 80°C/min temperature elevation rate, 1200°C final temperature, and a 20-minute dwell time at the final temperature. A pilot study was conducted to determine a final temperature that would yield a smooth and aesthetically pleasing appearance.

**Figure 1:**
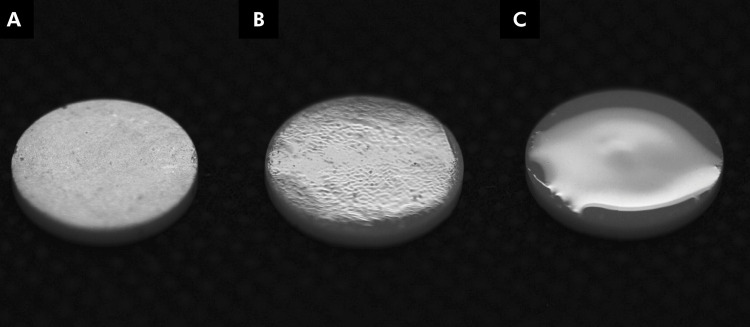
Final appearance of the 3Y-TZP specimens: as-sintered (a), coated with glaze (b), and coated with borosilicate glass (c).

A previously trained operator performed the glaze and experimental glass applications. The final thickness of the specimens was measured using a digital caliper [G: 1.39 (0.05) mm, SL: 1.37 (0.05) mm]. The appearance of the specimens from each group is depicted in Figure 1 .

### Roughness analysis

All the specimens from each group (n = 30) were analyzed using a contact roughness tester (SJ 400, Mitutoyo, Tokyo, Japan). Three equidistant parallel measurements were taken for each specimen at a speed of 0.2 mm/s, and an additional three parallel measurements were taken with the same specimen rotated by 90 degrees. The analysis adhered to ISO 4287-1997 standards, employing a Gaussian filter and a cut-off wavelength value of 0.8 mm. Average values were computed for each sample, and the mean Ra (average roughness) and Rz (ten-point-mean roughness) values were utilized in the subsequent statistical analysis.

### X-ray diffraction analysis (XRD)

Two specimens from each group were analyzed using X-ray diffractometry to identify the crystalline phases. Cu Kα radiation with a wavelength of 0.15418 nm was employed within a range (-2) spanning 20° to 40°. The scanning parameters were a step of 10.1600 s, a step size of 0.0170°, scanning speed of 2°min^-1^, 40 kV, and 40 mA.

The XRD data underwent analysis by identifying the crystalline phases after comparing the experimental spectra with standard diffraction spectra from the JCPDS (Joint Committee on Powder Diffraction Standards) and ICSD (Inorganic Crystal Structure). The High Score software program (Philips X’pert PANalytical, Almelo, The Netherlands) was utilized to obtain the spectral attributions.

### Color and translucency measurement

The samples (n = 30) were assessed using a spectrophotometer (VITA Easyshade, Vita Zahnfabrik, Bad Säckingen, Germany). CIE L*a*b* coordinates (International Commission on Illumination) were recorded, where L* represents the luminosity axis with values ranging from 0 (black) to 100 (white), and a* and b* denote the color coordinates along the green-red and blue-yellow axes, respectively. Measurements were taken over black (L* = 27.94, a* = -0.01, b* = 0.03), white (L* = 92.95, a* = 0.78, b* = 3.57), and gray (L* = 50.30, a* = -1.41, b* = -2.37) backgrounds. The observer angle was set at 10°, and the chosen illuminant was D65. A coupling solution (glycerol, C_3_H_8_O_3_, Vetec Química Fina Ltda, Rio de Janeiro, Brazil) was used between the specimen and the background to minimize light scattering.

Each specimen underwent three measurements, and the average L*a*b* values were utilized in the translucency and color difference calculations. The color differences (∆E_00_) between the control and experimental groups were computed using CIEDE 2000 (Eq. 1) based on measurements taken over a gray background. For clinical interpretation, the perceptibility (∆E_00_ > 0.8) and unacceptability (∆E_00_ > 1.8) thresholds established by Paravina et al. (2019) were taken into consideration.^20^


$$\Delta E_{00}={\left[{\left(\frac{\Delta C^{\prime }}{K_{L}S_{L}}\right)}^{2}+{\left(\frac{\Delta H^{\prime }}{K_{H}S_{H}}\right)}^{2}+R_{T}\left(\frac{\Delta C^{\prime }}{K_{C}S_{C}}\right)\left(\frac{\Delta H^{\prime }}{K_{H}S_{H}}\right)\right]}^{1/2}$$ Eq. (1)

where ∆L’, ∆C’ and ∆H’ are the differences in luminosity, chroma, and hue, respectively, for a pair of measurements; RT is a rotation function accounting for the interaction between chroma and hue differences in the blue region; SL, SC, and SH are weighting functions adjusting the total color difference for variations in the location of the color difference pair in L*, a*, b* coordinates; and kL, kC, and kH are the parametric factors serving as correction terms for deviation from reference experimental conditions.

Measurements conducted over black and white backgrounds were employed to calculate the translucency parameter (TP_00_) for each experimental group using CIEDE 2000 (Eq. 1). The simple translucency difference (∆TP_00_) between the experimental groups (SL or G) and the control group was computed for clinical interpretation, where ∆TP_00_ > 0.62 was considered the perceptibility threshold, and ∆TP_00_ > 2.62 was considered the unacceptability threshold.^21^

### Biaxial flexural strength

The biaxial flexural strength test (n = 30) was conducted using a piston-on-three-ball setup (ISO 6872/2015). The samples were positioned with the coated surface on a circular base featuring three equidistant metallic spheres (Æ = 3.2 mm), forming a plane. The load was applied with a flat-tip piston (Æ = 1.6 mm) fixed on a universal testing machine (EMIC DL-1000, EMIC, Sao Jose dos Pinhais, Brazil) equipped with a load cell (1.000 kgf). The load was applied at a constant speed of 1 mm/min until fracture. All the tests were conducted under water. The biaxial flexural strength (σ, MPa) of the discs was determined using Eq. (2).


$$\sigma =-0.2387P\left(X-\frac{Y}{d^{2}}\right)$$ Eq. (2)

where P is the load (N), X and Y are the parameters related to the elastic properties of the material [Poisson’s ratio (0.3) and elastic modulus (210 GPa)], and d is the thickness of the specimen at the fracture origin (mm).

The fractured specimens were inspected using a stereomicroscope (Discovery V20, Carl Zeiss, Jena, Thuringia, Germany), and representative specimens (n = 1) were further evaluated in a scanning electron microscope (SEM) to determine the failure origin and characteristics (fractography).

### Fracture toughness

An additional set of specimens (n = 10) was prepared to assess fracture toughness. Five Vickers indentations were made with varying loads (C group, 9.8 N for 15 s; G group, 4.9 N for 15 s; SL group, 0.0987 N for 15 s) using an HMV-G21 instrument (Shimadzu, Kyoto, Japan). The length of the developed cracks was measured, and fracture toughness was estimated using Eq. (3), with the respective control values incorporated into the calculations.


$$K_{Ic}==k{\left(\frac{E}{H}\right)}^{0.5}\times \frac{P}{c^{3/2}}$$ Eq. (3)

where E is the elastic modulus, P is the applied load (in N), H is the Vickers hardness, given by H = 1.8544P/a^2^ (in GPa), c is the average radial crack length measured from the center of the indentation (in m), and k is a constant equal to 0.016. The E values used for each group were C = 200 GPa,^22^ G = 60 GPa,^23^ and SL = 51 GPa.^24^

### Statistical analysis

The statistical analysis was conducted using SigmaPlot 12.0 software (Systat Software Inc., San Jose, USA), and a significance level of 5%. Prior to analysis, the data were assessed for normality using the Shapiro-Wilk test, and for homoscedasticity using the Levene test. One-way ANOVA was employed to analyze flexural strength, translucency, roughness, and fracture toughness. A multiple comparison analysis was performed using Tukey’s tests. Color difference data were analyzed using the t-test. Additionally, a Weibull analysis was applied to the flexural strength data, where Weibull’s modulus and characteristic strength were obtained through a maximum likelihood estimation using Minitab 16 software (Minitab Inc., State College, USA), and a 95% confidence interval.

## Results

**Figure 2:**
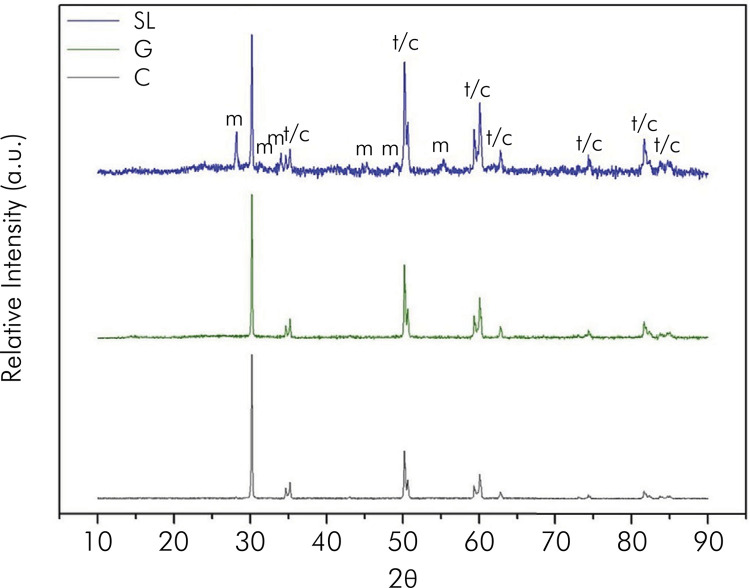
X-ray diffractograms from the study groups. The peaks represent tetragonal zirconia (t), cubic zirconia (c), and monoclinic zirconia (m).

The roughness results (Ra and Rz) are presented in Table 2 . Borosilicate glass specimens had significantly lower values of Ra and Rz compared to specimens from the glaze and as-sintered groups (p < 0.001). The XRD analysis ( Figure 2 ) demonstrated peaks characteristic of tetragonal zirconia in all groups. The presence of the cubic and monoclinic phases was observed exclusively in the SL group.

**Table 2:** Average roughness (Ra), ten-point-mean roughness (Rz), fracture toughness (KIc), biaxial flexural strength (BFS), Weibull modulus (m), and characteristic strength (σΘ) means, standard deviations, and confidence intervals (CI) obtained for the study groups. Different letters within a column indicate statistically significant differences among the study groups (p < 0.05, one-way ANOVA and Tukey’s test).

Groups	Ra (µm)	Rz (µm)	K_Ic_ (MPa m^0.5^)	BFS (MPa)	m CI (95%)	σθ CI (95%)
C	0.30^A^ (0.03)	2.13^A^ (0.20)	5.50^A^ (0.44)	859.41^A^ (157.57)	6.3^A^ (5.0–8.1)	920.8^A^ (865.7–979.3)
G	0.08^B^ (0.07)	0.43^B^ (0.31)	1.31^B^ (0.27)	816.0 ^A^ (97.22)	10.1^AB^ (8.1–12.6)	855.4^A^ (822.7–889.4)
SL	0.03^C^ (0.01)	0.18^C^ (0.10)	1.51^B^ (0.15)	1,025.8^B^ (122.85)	10.8^AB^ (8.6–13.4)	1072.2^B^ (1,033.1–1,113)

Table 3 outlines the translucency and color difference results obtained for the study groups. The translucency measurements for the control group were higher than those of the experimental groups (p < 0.001), which were similar to each other. However, the translucency differences of G and SL compared to C were not clinically perceptible. The color difference produced by glaze and borosilicate glass was statistically similar (p = 0.116) and clinically perceptible.

**Table 3:** Color difference (ΔE00), translucency (TP00), and translucency difference (ΔTP00) means and standard deviations obtained for the study groups.

Groups	∆E_00_	Acceptable match^*^	TP_00_	∆TP_00_	Acceptable match^**^
C	-	-	11.78^A^ (0.67)	-	-
G	3.44^A^ (0.64)	Yes	10.76^B^ (0.63)	1.02	Yes
SL	3.78^A^ (0.70)	Yes	10.69^B^ (0.96)	1.09	Yes

Different letters within a column indicate statistically significant differences among the study groups (p < 0.05, t-test for ΔE_00_, and one-way ANOVA and Tukey’s test for TP_00_). ∆Eab, color difference between G and SL groups. * Acceptable match if ∆Eab ≤1.2.^20^ ** Acceptable match if ∆TP00 >0.6, ≤2.6.^20^

**Figure 3:**
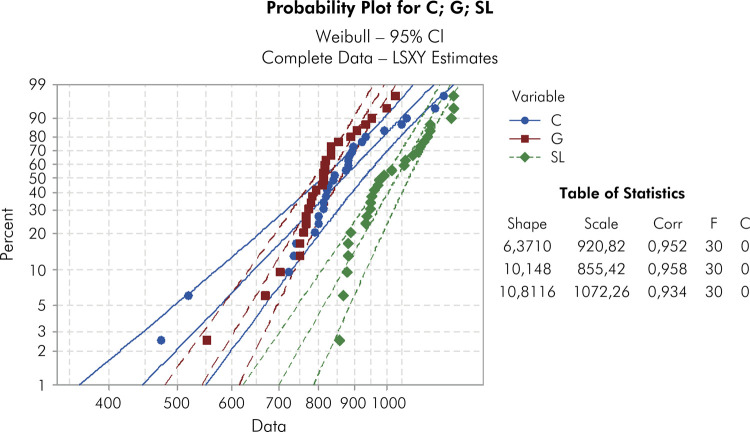
Weibull distribution graphs for groups C, G, and SL.

The flexural strength results are shown in Table 2 . The as-sintered and glaze groups had similar strength values (p > 0.005). The application of borosilicate led to the highest biaxial flexural strength values (p < 0.001). The characteristic strength of SL (1087) was significantly different compared to that of C (835.2) and G (852.3). However, the Weibull modulus of G (10.1) and SL (10.8) were similar ( Table 2 ). Figure 3 illustrates the failure probability plot, highlighting the superior behavior of the SL group compared to the C group.

**Figure 4:**
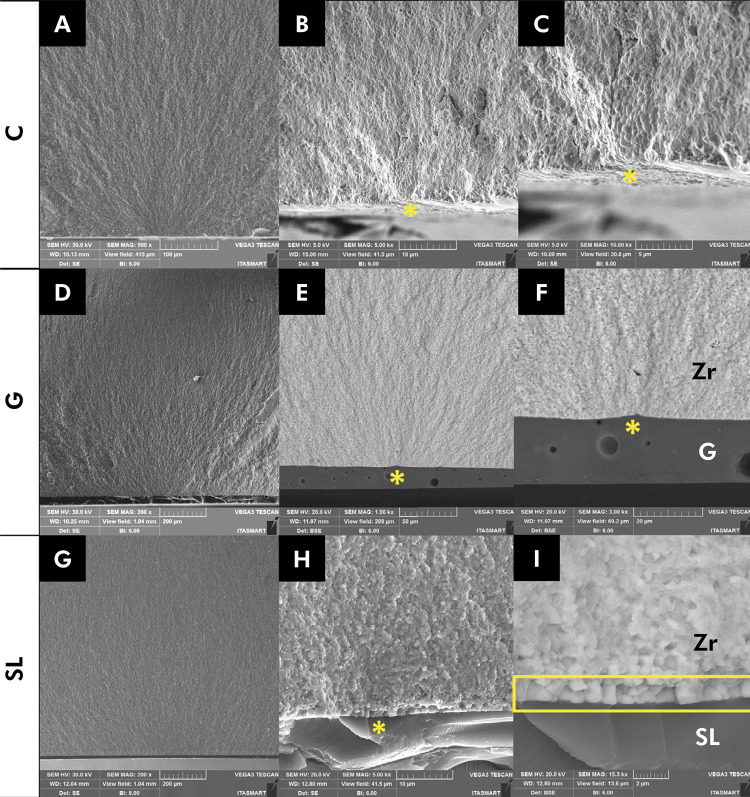
SEM images of the fractured surfaces according to the study groups. Group C (a, 500x; b, 5000x; and c, 10000x). Group G (d, 200x; e, 1000x; and f, 3000x). Group SL (g, 200x; h, 5000x, and I, 15000x). The origin of the fracture is indicated by a yellow asterisk. Monoclinic grains formed on the interface between borosilicate glass and zirconia are indicated by a yellow rectangle. Zr, zirconia; G, glaze; SL, borosilicate glass. All fractures originated on the tensile side of the specimens.

Figure 4 depicts the fractographic analysis. The fracture origin for all groups was identified on the zirconia, as highlighted by the yellow asterisk. A backscattered micrograph is displayed in Figure 4 , e and f. Zirconia appears denser and is represented by a lighter color, whereas glaze can be identified by darker colors. The cross-section view revealed porosities in the glaze layer ( Figure 4 , e and f), whereas the borosilicate glass exhibited a more homogeneous glass layer ( Figure 4 , h and i).

Statistical differences in fracture toughness were observed among all groups (p < 0.001, Table 2 ). The values from the control group (as-sintered) were the highest, and were significantly different from those from both the glaze (G) and borosilicate glass (SL) groups.

## Discussion

This study assessed the mechanical and optical characteristics of alternative surface finishings for dental zirconia. Borosilicate glass provided specimens with the highest flexural strength and smoothest surface, but did not contribute to an increase in fracture toughness. Additionally, its optical behavior was similar to that of the commercial glaze. Consequently, the tested hypothesis was partially accepted.

Borosilicate glass improved the flexural strength of 3Y-TZP by approximately 19%. Boron concentrations of around 3% (as utilized in this study) have been reported to enhance the elastic modulus and hardness of silicate glasses, thereby potentially increasing strength.^25^ As highlighted by Campos et al.^8^ , the high sintering temperature for silicate glass can facilitate the movement of zirconia grains and fill defects in the ceramic’s microstructure. Additionally, boron has been reported in the literature as a component that decreases material viscosity, contributing to the low roughness values (Ra and Rz) observed for the SL group ( Figure 1 , Table 2 ).^26^ Even with the extended firing, the commercial glaze resulted in flexural strength similar to that observed for the as-sintered control group, confirming the findings from previous studies ( Table 2 ).^11^

The Vickers indentation method was chosen for fracture toughness calculation because it could detect surface differences caused by thin layers of glaze or experimental glass. Fracture toughness significantly decreased when glaze and borosilicate were applied. This decrease was attributed to the load being applied on the glaze and borosilicate glass, which are weaker than zirconia. 3Y-TZP displays a toughness mechanism known as martensitic transformation.^27^ When a crack develops in the material, the tetragonal grains transform into monoclinic, leading to an increase in volume by 3% to 5%. This growth prevents the crack from growing and, consequently, resists its propagation.^27 , 28^ As a result, the values of flexural strength and hardness are substantially increased compared to other ceramic materials ( Table 2 ).^29^ However, when a glaze layer is applied onto 3Y-TZP, the surface toughness drastically drops due to the lack of the phase transformation effect. On the other hand, borosilicate glass led to slightly higher toughness values than those of the commercial glaze ( Table 2 ). These results might be related to the compressive stresses caused by the lower CTE of the experimental glass (approximately 3.5 × 10–6 K^-1^) compared to that of zirconia (approximately 10.8 × 10–6 K^-1^).

The borosilicate glass interacted with the first layer of grains due to its wettability, inducing the transformation of tetragonal grains into monoclinic grains (Figures 2 and 4). This transformation is associated with the XRD results, in that this analysis showed the formation of the monoclinic phase ( Figure 2 ). The presence of monoclinic grains facilitates crack compression, consequently increasing biaxial flexural strength, as observed in our results.^18^ This process is akin to hydrothermal degradation, where, at high temperatures, the glass (in a liquid state) acts similarly to water, penetrating the zirconia microstructure and inducing the tetragonal-to-monoclinic phase transformation ( Figure 4 , g and h).^13^ This phenomenon was also observed in the study by Campos et al.^8^ . The authors developed an in-house glass with a composition similar to that of the present study, and compressive stress formation was observed in the group with glass due to the transformation of tetragonal to monoclinic phase zirconia. The XRD pattern of the borosilicate glass group also revealed the presence of the cubic phase. Zirconias with a higher cubic content typically exhibit lower strength.^22 , 30^ However, the low cubic content triggered by the application of borosilicate glass was not sufficient to diminish the flexural strength ( Figure 2 ).

The Weibull graph indicated no statistical difference between the G and SL groups ( Figure 3 ). Borosilicate glass and glaze exhibited a homogeneous microstructural behavior, minimizing surface defects ( Figure 4 , e through h), resulting in similar Weibull modulus variability, in line with the findings by Campos et al.^19^

The fractographic analysis revealed that the failure origin was in the zirconia surface for all groups ( Figure 4 ). Backscattered images were captured to identify the materials. Commercial glaze and borosilicate glass are distinguishable since their atomic weight is lower than that of zirconia.^13^ Since zirconia is denser, it is represented by a lighter color, whereas both glasses are displayed in darker colors. In a cross-section view, the glaze group exhibited bubbles within the glaze layer ( Figure 4 , e and f), which were not observed in the SL group. For the borosilicate glass group, the fracture occurred due to detachment of monoclinic grains located at the interface between zirconia and borosilicate glass, as shown in Figure 4 , h and i.

Cubic grains enable a more uniform emission of incident light due to their isotropic characteristics.^31^ The way light interacts with cubic grains results in higher translucency. Even with the detection of the cubic phase in the SL group, it was not sufficient to affect translucency, since TP_00_ values were similar when borosilicate glass or glaze were applied. Both SL and G led to significantly lower translucency than the control, which can be attributed to the increase in thickness when the glassy layer was applied. Despite the statistical difference, the TP_00_ differences between SL or G and the control fall within the acceptable match category defined by Paravina et al.^20^ (∆TP_00_ > 0.6, ≤ 2.6; Table 3 ).

On the other hand, the color differences observed in SL and G were statistically similar, and the difference between them corresponded to an excellent match (∆Eab ≤ 1.2), as confirmed by Paravina et al.^20^ ( Table 3 ). Figure 1 shows how the glassy surface changed the final appearance of the material. Light scatters more at the 3Y-TZP grain boundaries than at the glass matrix. The change in light interaction when a glassy material is applied over zirconia results in higher luminosity and color alteration. However, the color difference caused by borosilicate glass was similar to that caused by the commercial glaze, indicating the feasibility of the experimental glass.

This is an inaugural investigation on an alternative glass for the finishing of monolithic 3Y-TZP restorations. Our findings indicate that the material tested is promising. Finishing treatments capable of enhancing the mechanical properties of the materials are considered advantageous. Nonetheless, further investigations into wear and fatigue, specifically involving crown-shaped specimens, are recommended to validate the viability of utilizing borosilicate glass.

## Conclusion

Borosilicate glass imparted a smoother surface to 3Y-TZP specimens compared to commercial glaze, and application of this experimental glass resulted in an enhancement of specimen biaxial flexural strength. Moreover, the influence of borosilicate glass on the optical properties of 3Y-TZP was similar to that of a commercial glaze.
